# 3-(4-Bromo­phenyl­sulfon­yl)-5-cyclo­pentyl-2-methyl-1-benzofuran

**DOI:** 10.1107/S1600536812019411

**Published:** 2012-05-05

**Authors:** Hong Dae Choi, Pil Ja Seo, Uk Lee

**Affiliations:** a Department of Chemistry, Dongeui University, San 24 Kaya-dong Busanjin-gu, Busan 614-714, Republic of Korea; b Department of Chemistry, Pukyong National University, 599-1 Daeyeon 3-dong, Nam-gu, Busan 608-737, Republic of Korea

## Abstract

In the title compound, C_20_H_19_BrO_3_S, the cyclo­pentyl ring adopts an envelope conformation. The 4-bromo­phenyl ring makes a dihedral angle of 82.09 (6)° with the mean plane [mean deviation = 0.026 (2) Å] of the benzofuran fragment. In the crystal, mol­ecules are linked by weak C—H⋯O hydrogen bonds and Br⋯O contacts [3.309 (2) Å].

## Related literature

For background information and the crystal structure of a related compound, see: Seo *et al.* (2011 ▶). For a review of halogen bonding, see: Politzer *et al.* (2007 ▶).
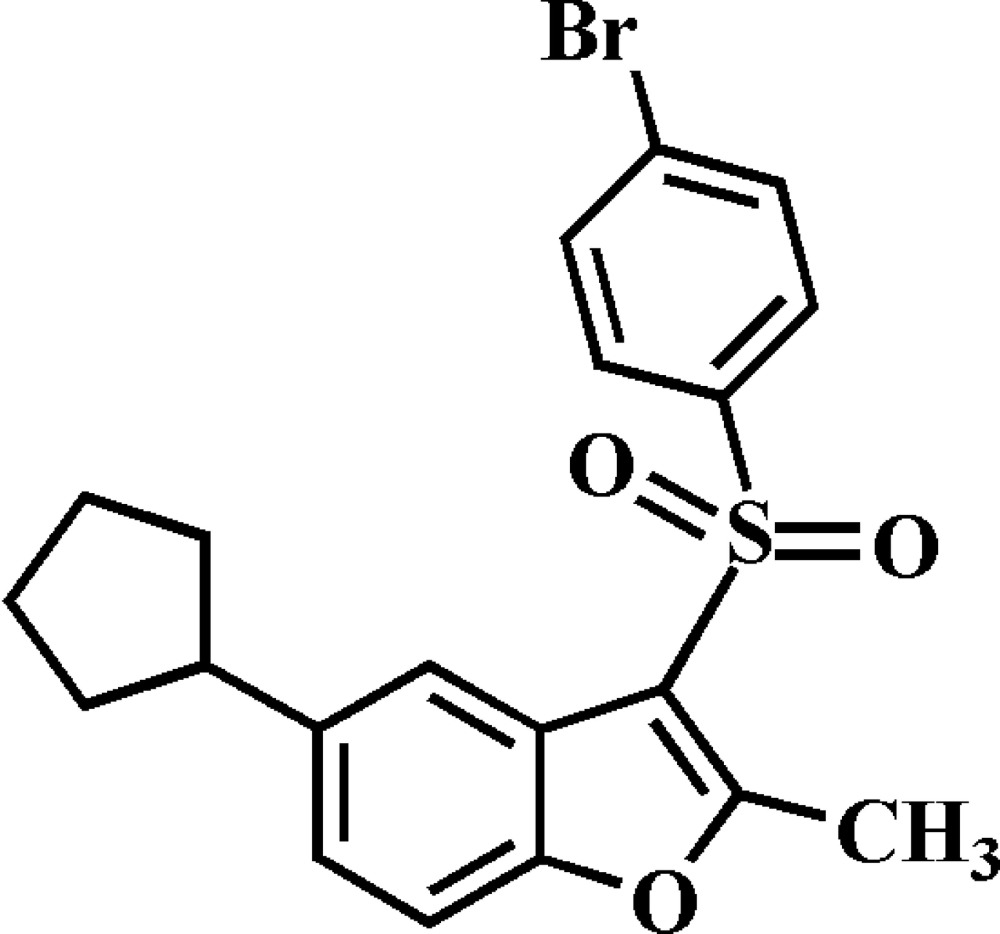


## Experimental

### 

#### Crystal data

C_20_H_19_BrO_3_S
*M* *_r_* = 419.32
Monoclinic, 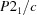

*a* = 6.8956 (2) Å
*b* = 17.1034 (3) Å
*c* = 15.7773 (3) Å
β = 97.533 (1)°
*V* = 1844.69 (7) Å^3^
*Z* = 4
Mo *K*α radiation
μ = 2.36 mm^−1^
*T* = 173 K
0.38 × 0.34 × 0.27 mm

#### Data collection

Bruker SMART APEXII CCD diffractometer
Absorption correction: multi-scan (*SADABS*; Bruker, 2009 ▶) *T*_min_ = 0.469, *T*_max_ = 0.570
18310 measured reflections
4602 independent reflections
3416 reflections with *I* > 2σ(*I*)
*R*_int_ = 0.041

#### Refinement

*R*[*F* ^2^ > 2σ(*F* ^2^)] = 0.038
*wR*(*F* ^2^) = 0.093
*S* = 1.02
4602 reflections
227 parameters
H-atom parameters constrained
Δρ_max_ = 0.58 e Å^−3^
Δρ_min_ = −0.73 e Å^−3^

### 

Data collection: *APEX2* (Bruker, 2009 ▶); cell refinement: *SAINT* (Bruker, 2009 ▶); data reduction: *SAINT*; program(s) used to solve structure: *SHELXS97* (Sheldrick, 2008 ▶); program(s) used to refine structure: *SHELXL97* (Sheldrick, 2008 ▶); molecular graphics: *ORTEP-3* (Farrugia, 1997 ▶) and *DIAMOND* (Brandenburg, 1998 ▶); software used to prepare material for publication: *SHELXL97*.

## Supplementary Material

Crystal structure: contains datablock(s) global, I. DOI: 10.1107/S1600536812019411/fy2055sup1.cif

Structure factors: contains datablock(s) I. DOI: 10.1107/S1600536812019411/fy2055Isup2.hkl

Supplementary material file. DOI: 10.1107/S1600536812019411/fy2055Isup3.cml

Additional supplementary materials:  crystallographic information; 3D view; checkCIF report

## Figures and Tables

**Table 1:** Hydrogen-bond geometry (Å, °)

*D*—H⋯*A*	*D*—H	H⋯*A*	*D*⋯*A*	*D*—H⋯*A*
C17—H17⋯O2^i^	0.95	2.51	3.169 (3)	126

Symmetry code: (i) 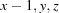.

**Fig. 1.**
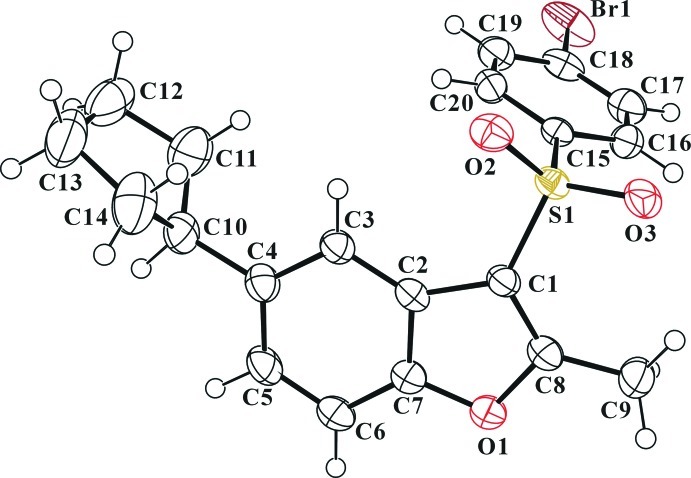
The molecular structure of the title compound with the atom numbering scheme. Displacement ellipsoids are drawn at the 50% probability level. H atoms are presented as small spheres of arbitrary radius.

**Fig. 2.**
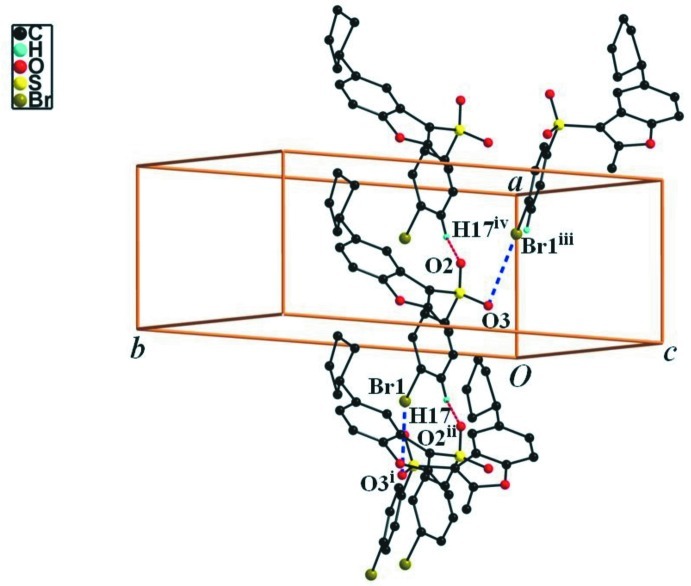
A view of the C—H···O and Br···O interactions (dotted lines) in the crystal structure of the title compound. H atoms not participating in hydrogen-bonding were omitted for clarity. [Symmetry codes: (i) x - 1, - y + 1/2, z - 1/2; (ii) x + 1, y, z; (iii) x + 1, - y + 1/2, z + 1/2; (iv) x + 1, y, z.]

## supplementary crystallographic information

### Comment

As a part of our ongoing study of 5-cyclopentyl-2-methyl-1-benzofuran derivatives containing a 3-phenylsulfonyl substituent (Seo *et al.*, 2011), we report herein the crystal structure of the title compound.

In the title molecule (Fig. 1), the benzofuran unit is essentially planar, with a mean deviation of 0.026 (2) Å from the least-squares plane defined by the nine constituent atoms. The cyclopentyl ring has an envelope conformtion. The dihedral angle between the 4-bromophenyl ring and the mean plane of the benzofurn fragment is 82.09 (6)°. In the crystal structure (Fig. 2), molecules are connected by weak C—H···O interactions (Table 1). The crystal packing (Fig. 2) also exhibits Br···O halogen-bonding interactions between the bromine atom and the O atom of the O═S═O unit [Br1···O3^i^ = 3.309 (2) Å, C18—Br1···O3^i^ = 153.82 (8)°] (Politzer *et al.*, 2007).

### Experimental

3-Chloroperoxybenzoic acid (77%, 381 mg, 1.7 mmol) was added in small portions to a stirred solution of 3-(4-bromophenylsulfanyl)-5-cyclopentyl-2-methyl-1-benzofuran (310 mg, 0.8 mmol) in dichloromethane (30 mL) at 273 K. After being stirred at room temperature for 10 h, the mixture was washed with saturated sodium bicarbonate solution and the organic layer was separated, dried over magnesium sulfate, filtered and concentrated at reduced pressure. The residue was purified by column chromatography (benzene) to afford the title compound as a colorless solid [yield 71%, m.p. 423–424 K; *R*_f_ = 0.48 (benzene)]. Single crystals suitable for X-ray diffraction were prepared by slow evaporation of a solution of the title compound in acetone at room temperature.

### Refinement

All H atoms were positioned geometrically and refined using a riding model, with C—H = 0.95 Å for the aryl, 1.00 Å for the methine, 0.99 Å for the methylene, and 0.98 Å for the methyl H atoms. *U*_iso_(H) = 1.2*U*_eq_(C) for the aryl, methine, and methylene H atoms, and 1.5*U*_eq_(C) for the methyl H atoms. The positions of methyl hydrogens were optimized rotationally.

### Crystal data

**Table:**

C_20_H_19_BrO_3_S	*F*(000) = 856
*M**_r_* = 419.32	*D*_x_ = 1.510 Mg m^−^^3^
Monoclinic, *P*2_1_/*c*	Mo *K*α radiation, λ = 0.71073 Å
Hall symbol: -P 2ybc	Cell parameters from 5006 reflections
*a* = 6.8956 (2) Å	θ = 2.4–26.9°
*b* = 17.1034 (3) Å	µ = 2.36 mm^−^^1^
*c* = 15.7773 (3) Å	*T* = 173 K
β = 97.533 (1)°	Block, colourless
*V* = 1844.69 (7) Å^3^	0.38 × 0.34 × 0.27 mm
*Z* = 4	

### Data collection

**Table:**

Bruker SMART APEXII CCD diffractometer	4602 independent reflections
Radiation source: rotating anode	3416 reflections with *I* > 2σ(*I*)
Graphite multilayer monochromator	*R*_int_ = 0.041
Detector resolution: 10.0 pixels mm^-1^	θ_max_ = 28.4°, θ_min_ = 1.8°
φ and ω scans	*h* = −9→9
Absorption correction: multi-scan (*SADABS*; Bruker, 2009)	*k* = −22→22
*T*_min_ = 0.469, *T*_max_ = 0.570	*l* = −21→19
18310 measured reflections	

### Refinement

**Table:**

Refinement on *F*^2^	Primary atom site location: structure-invariant direct methods
Least-squares matrix: full	Secondary atom site location: difference Fourier map
*R*[*F*^2^ > 2σ(*F*^2^)] = 0.038	Hydrogen site location: difference Fourier map
*wR*(*F*^2^) = 0.093	H-atom parameters constrained
*S* = 1.02	*w* = 1/[σ^2^(*F*_o_^2^) + (0.0389*P*)^2^ + 0.8231*P*] where *P* = (*F*_o_^2^ + 2*F*_c_^2^)/3
4602 reflections	(Δ/σ)_max_ < 0.001
227 parameters	Δρ_max_ = 0.58 e Å^−^^3^
0 restraints	Δρ_min_ = −0.73 e Å^−^^3^

### Special details

**Table:**

Geometry. All esds (except the esd in the dihedral angle between two l.s. planes) are estimated using the full covariance matrix. The cell esds are taken into account individually in the estimation of esds in distances, angles and torsion angles; correlations between esds in cell parameters are only used when they are defined by crystal symmetry. An approximate (isotropic) treatment of cell esds is used for estimating esds involving l.s. planes.
Refinement. Refinement of F^2^ against ALL reflections. The weighted R-factor wR and goodness of fit S are based on F^2^, conventional R-factors R are based on F, with F set to zero for negative F^2^. The threshold expression of F^2^ > 2sigma(F^2^) is used only for calculating R-factors(gt) etc. and is not relevant to the choice of reflections for refinement. R-factors based on F^2^ are statistically about twice as large as those based on F, and R- factors based on ALL data will be even larger.

### Fractional atomic coordinates and isotropic or equivalent isotropic displacement parameters (Å^2^)

**Table:**

	*x*	*y*	*z*	*U*_iso_*/*U*_eq_	
Br1	−0.32092 (5)	0.301064 (16)	0.016006 (19)	0.06550 (13)	
S1	0.31357 (7)	0.28508 (3)	0.34905 (3)	0.02688 (12)	
O1	0.2226 (2)	0.48448 (8)	0.45861 (10)	0.0398 (4)	
O2	0.5019 (2)	0.27163 (8)	0.32240 (10)	0.0356 (3)	
O3	0.2418 (2)	0.23232 (8)	0.40858 (9)	0.0363 (3)	
C1	0.3124 (3)	0.37925 (11)	0.38922 (12)	0.0282 (4)	
C2	0.4174 (3)	0.44557 (11)	0.36055 (13)	0.0288 (4)	
C3	0.5464 (3)	0.45853 (11)	0.30148 (13)	0.0313 (4)	
H3	0.5924	0.4161	0.2707	0.038*	
C4	0.6077 (3)	0.53476 (12)	0.28803 (14)	0.0351 (5)	
C5	0.5410 (4)	0.59592 (12)	0.33602 (15)	0.0406 (5)	
H5	0.5841	0.6476	0.3268	0.049*	
C6	0.4158 (4)	0.58411 (12)	0.39592 (15)	0.0408 (5)	
H6	0.3735	0.6259	0.4286	0.049*	
C7	0.3554 (3)	0.50843 (12)	0.40580 (14)	0.0338 (5)	
C8	0.1965 (3)	0.40590 (12)	0.44621 (14)	0.0351 (5)	
C9	0.0490 (4)	0.36946 (14)	0.49391 (18)	0.0509 (7)	
H9A	−0.0824	0.3813	0.4652	0.076*	
H9B	0.0634	0.3903	0.5523	0.076*	
H9C	0.0685	0.3127	0.4959	0.076*	
C10	0.7396 (4)	0.55251 (12)	0.22173 (16)	0.0426 (6)	
H10	0.7525	0.6106	0.2187	0.051*	
C11	0.6704 (5)	0.52353 (19)	0.13200 (18)	0.0642 (8)	
H11A	0.5648	0.5574	0.1037	0.077*	
H11B	0.6206	0.4693	0.1334	0.077*	
C12	0.8493 (6)	0.52695 (18)	0.0848 (2)	0.0763 (11)	
H12A	0.8563	0.4797	0.0491	0.092*	
H12B	0.8435	0.5736	0.0474	0.092*	
C13	1.0259 (6)	0.53131 (18)	0.1533 (3)	0.0822 (12)	
H13A	1.0902	0.5830	0.1525	0.099*	
H13B	1.1222	0.4902	0.1443	0.099*	
C14	0.9451 (4)	0.5187 (2)	0.2377 (2)	0.0685 (9)	
H14A	0.9416	0.4623	0.2519	0.082*	
H14B	1.0253	0.5465	0.2850	0.082*	
C15	0.1402 (3)	0.28833 (10)	0.25667 (12)	0.0257 (4)	
C16	−0.0527 (3)	0.26893 (12)	0.26253 (14)	0.0339 (5)	
H16	−0.0905	0.2530	0.3157	0.041*	
C17	−0.1896 (3)	0.27292 (13)	0.19020 (15)	0.0402 (5)	
H17	−0.3224	0.2596	0.1931	0.048*	
C18	−0.1312 (3)	0.29636 (12)	0.11402 (15)	0.0377 (5)	
C19	0.0603 (4)	0.31567 (12)	0.10699 (14)	0.0382 (5)	
H19	0.0971	0.3315	0.0536	0.046*	
C20	0.1983 (3)	0.31153 (11)	0.17929 (13)	0.0318 (4)	
H20	0.3311	0.3244	0.1760	0.038*	

### Atomic displacement parameters (Å^2^)

**Table:**

	*U*^11^	*U*^22^	*U*^33^	*U*^12^	*U*^13^	*U*^23^
Br1	0.0750 (2)	0.04795 (17)	0.0610 (2)	0.00540 (13)	−0.03790 (16)	−0.00334 (12)
S1	0.0311 (3)	0.0203 (2)	0.0284 (2)	0.00110 (17)	0.00070 (19)	−0.00012 (18)
O1	0.0528 (10)	0.0260 (7)	0.0438 (9)	−0.0022 (6)	0.0187 (8)	−0.0062 (6)
O2	0.0312 (8)	0.0323 (7)	0.0424 (9)	0.0064 (6)	0.0021 (7)	−0.0042 (6)
O3	0.0500 (10)	0.0247 (7)	0.0336 (8)	−0.0015 (6)	0.0034 (7)	0.0042 (6)
C1	0.0340 (11)	0.0227 (9)	0.0277 (10)	−0.0017 (8)	0.0030 (8)	−0.0015 (8)
C2	0.0317 (11)	0.0228 (9)	0.0306 (10)	−0.0021 (7)	−0.0001 (8)	−0.0017 (7)
C3	0.0334 (12)	0.0258 (10)	0.0349 (11)	−0.0007 (8)	0.0050 (9)	−0.0013 (8)
C4	0.0371 (13)	0.0282 (10)	0.0402 (12)	−0.0016 (8)	0.0057 (9)	0.0031 (9)
C5	0.0473 (14)	0.0229 (10)	0.0526 (14)	−0.0043 (9)	0.0098 (11)	−0.0004 (9)
C6	0.0527 (15)	0.0246 (10)	0.0466 (13)	−0.0029 (9)	0.0114 (11)	−0.0071 (9)
C7	0.0382 (13)	0.0291 (10)	0.0346 (11)	−0.0018 (8)	0.0068 (9)	−0.0031 (8)
C8	0.0435 (13)	0.0246 (10)	0.0377 (12)	−0.0023 (8)	0.0072 (9)	−0.0026 (8)
C9	0.0638 (18)	0.0349 (12)	0.0610 (16)	−0.0046 (11)	0.0349 (14)	−0.0037 (11)
C10	0.0514 (15)	0.0244 (10)	0.0551 (15)	−0.0008 (9)	0.0188 (12)	0.0070 (10)
C11	0.076 (2)	0.0694 (19)	0.0507 (17)	−0.0048 (15)	0.0195 (15)	0.0125 (14)
C12	0.113 (3)	0.0466 (16)	0.082 (2)	0.0057 (17)	0.061 (2)	0.0127 (15)
C13	0.075 (2)	0.0528 (18)	0.132 (3)	−0.0021 (16)	0.065 (3)	0.0032 (19)
C14	0.0474 (18)	0.072 (2)	0.088 (2)	−0.0004 (14)	0.0185 (16)	0.0145 (17)
C15	0.0281 (11)	0.0196 (8)	0.0291 (10)	0.0022 (7)	0.0027 (8)	−0.0020 (7)
C16	0.0303 (12)	0.0347 (11)	0.0378 (12)	−0.0025 (8)	0.0083 (9)	−0.0032 (9)
C17	0.0283 (12)	0.0408 (12)	0.0502 (14)	−0.0009 (9)	0.0006 (10)	−0.0085 (10)
C18	0.0420 (14)	0.0276 (10)	0.0389 (12)	0.0057 (8)	−0.0117 (10)	−0.0069 (9)
C19	0.0542 (15)	0.0315 (11)	0.0279 (11)	−0.0023 (9)	0.0014 (10)	0.0011 (8)
C20	0.0332 (12)	0.0295 (10)	0.0329 (11)	−0.0035 (8)	0.0041 (9)	−0.0007 (8)

### Geometric parameters (Å, º)

**Table:**

Br1—C18	1.892 (2)	C10—C11	1.517 (4)
Br1—O3^i^	3.3090 (16)	C10—C14	1.521 (4)
S1—O2	1.4348 (15)	C10—H10	1.0000
S1—O3	1.4362 (15)	C11—C12	1.523 (4)
S1—C1	1.7313 (19)	C11—H11A	0.9900
S1—C15	1.760 (2)	C11—H11B	0.9900
O1—C8	1.367 (2)	C12—C13	1.520 (5)
O1—C7	1.379 (3)	C12—H12A	0.9900
C1—C8	1.358 (3)	C12—H12B	0.9900
C1—C2	1.449 (3)	C13—C14	1.525 (4)
C2—C3	1.388 (3)	C13—H13A	0.9900
C2—C7	1.388 (3)	C13—H13B	0.9900
C3—C4	1.395 (3)	C14—H14A	0.9900
C3—H3	0.9500	C14—H14B	0.9900
C4—C5	1.404 (3)	C15—C16	1.386 (3)
C4—C10	1.504 (3)	C15—C20	1.392 (3)
C5—C6	1.376 (3)	C16—C17	1.384 (3)
C5—H5	0.9500	C16—H16	0.9500
C6—C7	1.375 (3)	C17—C18	1.377 (3)
C6—H6	0.9500	C17—H17	0.9500
C8—C9	1.480 (3)	C18—C19	1.380 (4)
C9—H9A	0.9800	C19—C20	1.388 (3)
C9—H9B	0.9800	C19—H19	0.9500
C9—H9C	0.9800	C20—H20	0.9500
			
C18—Br1—O3^i^	153.82 (8)	C14—C10—H10	107.4
O2—S1—O3	119.56 (9)	C10—C11—C12	105.5 (3)
O2—S1—C1	107.71 (9)	C10—C11—H11A	110.6
O3—S1—C1	109.06 (9)	C12—C11—H11A	110.6
O2—S1—C15	107.74 (9)	C10—C11—H11B	110.6
O3—S1—C15	107.85 (9)	C12—C11—H11B	110.6
C1—S1—C15	103.80 (9)	H11A—C11—H11B	108.8
C8—O1—C7	106.92 (16)	C13—C12—C11	106.2 (3)
C8—C1—C2	107.78 (17)	C13—C12—H12A	110.5
C8—C1—S1	125.88 (16)	C11—C12—H12A	110.5
C2—C1—S1	125.97 (15)	C13—C12—H12B	110.5
C3—C2—C7	119.27 (18)	C11—C12—H12B	110.5
C3—C2—C1	136.45 (18)	H12A—C12—H12B	108.7
C7—C2—C1	104.23 (18)	C12—C13—C14	105.3 (3)
C2—C3—C4	119.00 (19)	C12—C13—H13A	110.7
C2—C3—H3	120.5	C14—C13—H13A	110.7
C4—C3—H3	120.5	C12—C13—H13B	110.7
C3—C4—C5	119.2 (2)	C14—C13—H13B	110.7
C3—C4—C10	121.13 (19)	H13A—C13—H13B	108.8
C5—C4—C10	119.70 (18)	C10—C14—C13	103.9 (3)
C6—C5—C4	122.7 (2)	C10—C14—H14A	111.0
C6—C5—H5	118.6	C13—C14—H14A	111.0
C4—C5—H5	118.6	C10—C14—H14B	111.0
C7—C6—C5	116.3 (2)	C13—C14—H14B	111.0
C7—C6—H6	121.9	H14A—C14—H14B	109.0
C5—C6—H6	121.9	C16—C15—C20	121.06 (19)
C6—C7—O1	125.68 (19)	C16—C15—S1	119.38 (16)
C6—C7—C2	123.5 (2)	C20—C15—S1	119.55 (15)
O1—C7—C2	110.74 (17)	C17—C16—C15	119.4 (2)
C1—C8—O1	110.30 (18)	C17—C16—H16	120.3
C1—C8—C9	134.34 (19)	C15—C16—H16	120.3
O1—C8—C9	115.33 (18)	C18—C17—C16	119.2 (2)
C8—C9—H9A	109.5	C18—C17—H17	120.4
C8—C9—H9B	109.5	C16—C17—H17	120.4
H9A—C9—H9B	109.5	C17—C18—C19	122.2 (2)
C8—C9—H9C	109.5	C17—C18—Br1	118.42 (18)
H9A—C9—H9C	109.5	C19—C18—Br1	119.40 (18)
H9B—C9—H9C	109.5	C18—C19—C20	118.8 (2)
C4—C10—C11	116.0 (2)	C18—C19—H19	120.6
C4—C10—C14	116.3 (2)	C20—C19—H19	120.6
C11—C10—C14	101.7 (2)	C19—C20—C15	119.4 (2)
C4—C10—H10	107.4	C19—C20—H20	120.3
C11—C10—H10	107.4	C15—C20—H20	120.3
			
O2—S1—C1—C8	−155.71 (19)	C7—O1—C8—C9	−176.6 (2)
O3—S1—C1—C8	−24.5 (2)	C3—C4—C10—C11	53.8 (3)
C15—S1—C1—C8	90.2 (2)	C5—C4—C10—C11	−124.7 (3)
O2—S1—C1—C2	32.2 (2)	C3—C4—C10—C14	−65.7 (3)
O3—S1—C1—C2	163.33 (17)	C5—C4—C10—C14	115.8 (3)
C15—S1—C1—C2	−81.9 (2)	C4—C10—C11—C12	−164.0 (2)
C8—C1—C2—C3	−176.0 (2)	C14—C10—C11—C12	−36.8 (3)
S1—C1—C2—C3	−2.7 (4)	C10—C11—C12—C13	18.2 (3)
C8—C1—C2—C7	1.1 (2)	C11—C12—C13—C14	7.8 (3)
S1—C1—C2—C7	174.40 (16)	C4—C10—C14—C13	168.5 (2)
C7—C2—C3—C4	−1.2 (3)	C11—C10—C14—C13	41.6 (3)
C1—C2—C3—C4	175.6 (2)	C12—C13—C14—C10	−30.8 (3)
C2—C3—C4—C5	1.5 (3)	O2—S1—C15—C16	153.86 (15)
C2—C3—C4—C10	−177.0 (2)	O3—S1—C15—C16	23.52 (18)
C3—C4—C5—C6	−0.4 (4)	C1—S1—C15—C16	−92.10 (17)
C10—C4—C5—C6	178.1 (2)	O2—S1—C15—C20	−27.16 (18)
C4—C5—C6—C7	−1.0 (4)	O3—S1—C15—C20	−157.50 (15)
C5—C6—C7—O1	−176.3 (2)	C1—S1—C15—C20	86.88 (17)
C5—C6—C7—C2	1.4 (4)	C20—C15—C16—C17	−0.2 (3)
C8—O1—C7—C6	177.0 (2)	S1—C15—C16—C17	178.79 (16)
C8—O1—C7—C2	−1.0 (2)	C15—C16—C17—C18	−0.2 (3)
C3—C2—C7—C6	−0.4 (3)	C16—C17—C18—C19	0.4 (3)
C1—C2—C7—C6	−178.1 (2)	C16—C17—C18—Br1	179.82 (16)
C3—C2—C7—O1	177.66 (19)	O3^i^—Br1—C18—C17	−10.6 (3)
C1—C2—C7—O1	−0.1 (2)	O3^i^—Br1—C18—C19	168.84 (12)
C2—C1—C8—O1	−1.8 (3)	C17—C18—C19—C20	−0.2 (3)
S1—C1—C8—O1	−175.07 (15)	Br1—C18—C19—C20	−179.62 (15)
C2—C1—C8—C9	176.1 (3)	C18—C19—C20—C15	−0.2 (3)
S1—C1—C8—C9	2.8 (4)	C16—C15—C20—C19	0.4 (3)
C7—O1—C8—C1	1.7 (2)	S1—C15—C20—C19	−178.60 (15)

Symmetry code: (i) *x*−1, −*y*+1/2, *z*−1/2.

### Hydrogen-bond geometry (Å, º)

**Table:**

*D*—H···*A*	*D*—H	H···*A*	*D*···*A*	*D*—H···*A*
C17—H17···O2^ii^	0.95	2.51	3.169 (3)	126

Symmetry code: (ii) *x*−1, *y*, *z*.
